# RADseq data reveal a lack of admixture in a mouse lemur contact zone contrary to previous microsatellite results

**DOI:** 10.1098/rspb.2022.0596

**Published:** 2022-08-10

**Authors:** Jelmer W. Poelstra, B. Karina Montero, Jan Lüdemann, Ziheng Yang, S. Jacques Rakotondranary, Paul Hohenlohe, Nadine Stetter, Jörg U. Ganzhorn, Anne D. Yoder

**Affiliations:** ^1^ Department of Biology, Duke University, Durham, NC 27708, USA; ^2^ Molecular and Cellular Imaging Center, Ohio State University, Wooster, OH 44691, USA; ^3^ Institute of Zoology, Department of Animal Ecology and Conservation, Universität Hamburg, Hamburg, 20146, Germany; ^4^ Department of Genetics, Evolution and Environment, University College London, London, UK; ^5^ Anthropobiologie et Développement Durable, Faculté des Sciences, Université d'Antananarivo, PO Box 906, Antananarivo 101, Madagascar; ^6^ Institute for Bioinformatics and Evolutionary Studies, Department of Biological Sciences, University of Idaho, Moscow, ID 83844, USA; ^7^ Bernhard Nocht Institute for Tropical Medicine, 20359 Hamburg, Germany

**Keywords:** RADseq, admixture, cryptic species, mouse lemurs, Madagascar, microsatellites

## Abstract

Microsatellites have been a workhorse of evolutionary genetic studies for decades and are still commonly in use for estimating signatures of genetic diversity at the population and species level across a multitude of taxa. Yet, the very high mutation rate of these loci is a double-edged sword, conferring great sensitivity at shallow levels of analysis (e.g. paternity analysis) but yielding considerable uncertainty for deeper evolutionary comparisons. For the present study, we used reduced representation genome-wide data (restriction site-associated DNA sequencing (RADseq)) to test for patterns of interspecific hybridization previously characterized using microsatellite data in a contact zone between two closely related mouse lemur species in Madagascar (*Microcebus murinus* and *Microcebus griseorufus*). We revisit this system by examining populations in, near, and far from the contact zone, including many of the same individuals that had previously been identified as hybrids with microsatellite data. Surprisingly, we find no evidence for admixed nuclear ancestry. Instead, re-analyses of microsatellite data and simulations suggest that previously inferred hybrids were false positives and that the program NewHybrids can be particularly sensitive to erroneously inferring hybrid ancestry. Combined with results from coalescent-based analyses and evidence for local syntopic co-occurrence, we conclude that the two mouse lemur species are in fact completely reproductively isolated, thus providing a new understanding of the evolutionary rate whereby reproductive isolation can be achieved in a primate.

## 1. Introduction

Microsatellites are tandem repeats of repetitive DNA that typically range in length from one to six nucleotides and occur at thousands of locations within the genomes of most organisms [1,2]. Individual microsatellite loci contain from as few as five to as many as 40 or more repeats, with copy number changes caused by slip-strand mispairing during DNA replication. Mutation rates for microsatellites are orders of magnitude higher than for other types of variants, including single nucleotide polymorphisms (SNPs), with the overall rate being a balance between the generation of replication errors and the correction of errors by proofreading and mismatch repair, all of which can vary by species [3]. Given their high rate of change, microsatellite loci have high allelic richness, often in excess of 10 alleles within humans and other primates [4]. This rich allelic diversity, combined with relatively low genotyping costs, have made microsatellites a popular genetic marker for applications ranging from paternity analysis to historical demography. In particular, they have proved useful for identifying conservation units in endangered species (e.g. [5]) as well as for revealing the presence of homoploid hybrid speciation (e.g. [6]).

Yet, their extreme sensitivity can also be cause for concern. The high rate of recurrent mutations (i.e. homoplasy) makes them poor indicators of long-term population history [2,4]. For example, the combination of homoplasy and potentially inappropriate models of mutational dynamics can yield highly inflated estimates of gene flow between populations and species [7,8]. Thus, inferences above all but the shallowest evolutionary levels should be treated with caution.

In this study, we revisit hypotheses of hybridization between two named species of mouse lemur, *Microcebus murinus* (*sensu lato*) and *Microcebus* *griseorufus*, reported from previous studies using microsatellite data [9–11]. These previous studies focused on two contact zones in the southeast of Madagascar wherein hybrids were reported to occur.

To date, seven different pairs of mouse lemur species have been shown to co-occur locally at various localities throughout Madagascar. One widespread species, *M. murinus*, is involved in five of these cases. In all but one of these seven cases of sympatry, no hybridization has been detected thus suggesting that co-occurring species are reproductively isolated. Sources of reproductive isolation among sympatric mouse lemurs are poorly known, but factors that may contribute to prezygotic isolation via differential mate choice may include divergence in acoustic [12,13] and olfactory signalling [14,15]. Additionally, opportunities for reproductive interaction may be reduced by ecological divergence manifesting, for example, in differential timing of the highly seasonal and temporally constrained reproductive season seen in mouse lemurs [16–18].

It is thus intriguing that hybridization has only been detected between *M. murinus* and *M. griseorufus*, using microsatellite loci [9,10], which is also unique among the seven cases of sympatry in consisting of a pair of sister lineages. Using the programs Structure [19] and GeneClass [20], Gligor *et al*. [9], p. 529) concluded that ‘most individuals within the transition zone’ had mixed ancestry (no individual-level assignments were made). Hapke *et al*. [10] studied a contact zone 40 km further north, and used the same set of microsatellite loci for a total of 159 mouse lemurs, with Structure and NewHybrids [21] identifying a total of 18 admixed individuals. Of these, 15 individuals showed signs of nuclear admixture (i.e. among microsatellites) whereas three had a mismatch between microsatellite and mitochondrial ancestry.

Here, we use restriction-site associated DNA sequencing (RADseq) data to revisit the contact zone area studied by Hapke *et al*. [10] and follow-up work in Lüdemann [11] that used the same microsatellites and methods. We have included a total of 130 individuals, including 18 of the individuals that were inferred to be hybrids by these studies in addition to samples from nearby and distant allopatric populations. To ensure that non-admixed individuals from parental species were present, as is critical for accurately identifying either the presence or absence of hybrids [22], we also include samples from nearby and distant allopatric populations. We examine individual-level admixture in the northern contact zone and used coalescent modelling to ask whether there is evidence for ongoing and/or ancestral gene flow between the species. To our surprise, we found no evidence for admixed individuals in the contact zone—including among the individuals previously identified as hybrids—and also infer a lack of ongoing gene flow between the two species more generally.

## 2. Methods

### (a) Sampling

Hapke *et al*. [10] and follow-up work in Lüdemann [11] detected hybridization between *M. murinus* (hereafter referred to as *murinus*) and *M. griseorufus* (hereafter referred to as *griseorufus*) using nine microsatellites and a fragment of the HV1 mitochondrial locus from individuals in the Andohahela area in southeastern Madagascar. We made use of a selection of 94 of their samples and augmented this dataset with 33 samples from distant, allopatric sites, and three *Microcebus rufus* samples that were used as an outgroup (electronic supplementary material, table S1, table S2).

**Figure 1.**
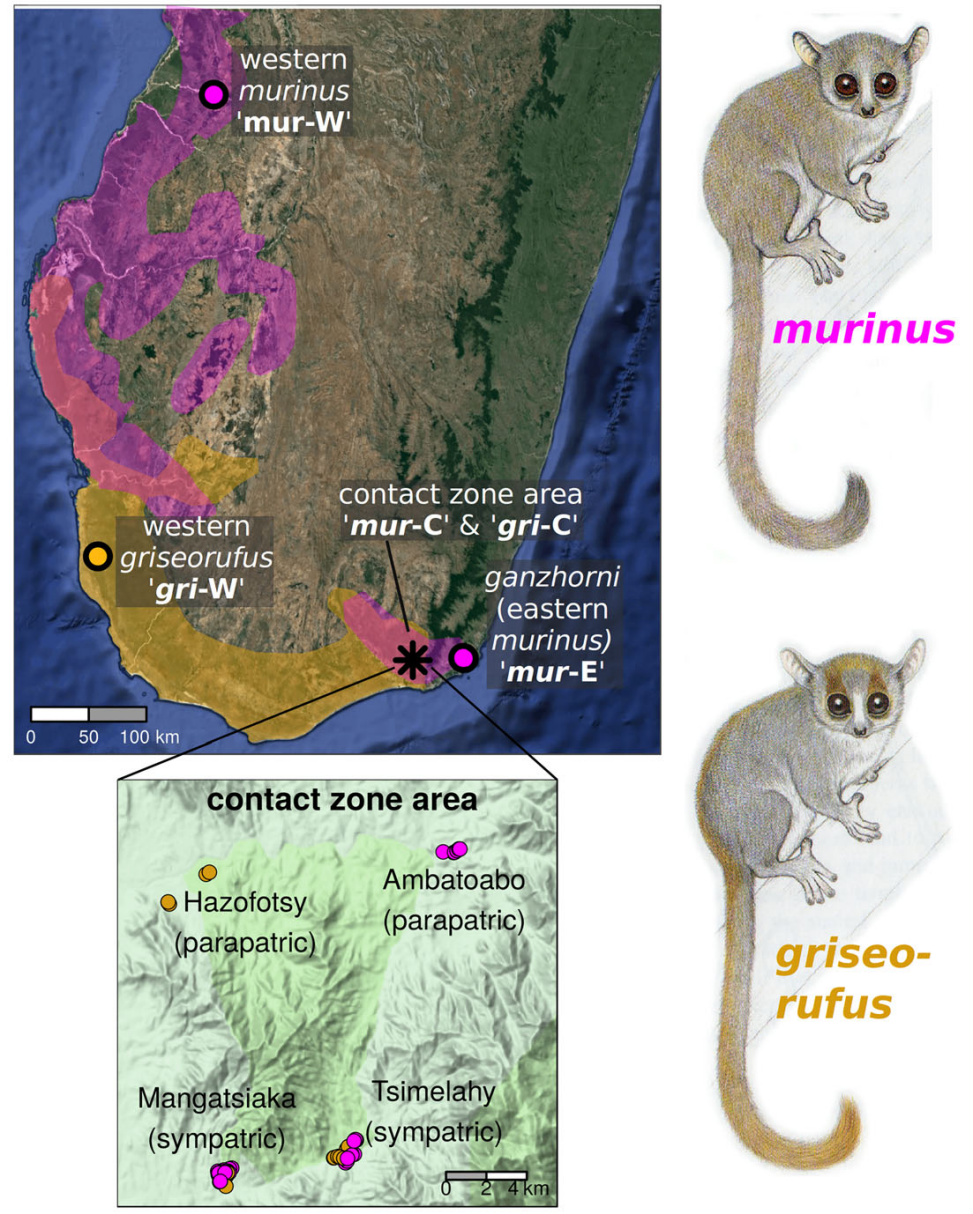
Distributions and sampling sites of *murinus* and *griseorufus* in southern Madagascar. The distribution of *murinus* is shown in purple and that of *griseorufus* in gold. A population in southeastern Madagascar was recently split from murinus as *M. ganzhorni*, but is here included within *murinus* *s.l.* The range of *M. murinus* extends to the north of the area shown in the map, whereas the entire distribution of *M. griseorufus* is shown. Inset: overview of sampling in the contact zone area (corresponding to the study site of [10]), showing two parapatric (Hazofotsy with *griseorufus* and Ambatoaba with *murinus*) and two sympatric (Mangatsiaka and Tsimelahy) sites. *Microcebus* illustrations courtesy of Stephen Nash. (Online version in colour.)

At two of the sites examined by Hapke *et al*. [10], they detected unadmixed individuals of both parental species as well as individuals with admixed ancestry (individuals inferred to be admixed by Hapke *et al*. [10] and Lüdemann [11] are hereafter referred to as ‘putative hybrids’). From these two contact zone sites, Mangatsiaka and Tsimelahy, which we refer to as ‘sympatric’ sites, we selected 78 samples (electronic supplementary material, table S1), including 15 individuals for which Hapke *et al*. [10] or Lüdemann [11] had detected nuclear admixture, and an additional three with a mitonuclear ancestry mismatch. We additionally selected samples from nearby sites at which Hapke *et al*. [10] had exclusively (or nearly so) detected unadmixed individuals of only one of the two species: eight *griseorufus* from Hazofotsy and eight *murinus* from Ambatoabo (electronic supplementary material, table S1). We refer to these contact zone sites as ‘parapatric’ sites. ‘Allopatric’ samples, taken well away from the contact zone, were represented by 14 *griseorufus*, eight *murinus* and 11 *Microcebus ganzhorni,* a species that was recently split from *murinus* [23]*,* from Mandena in far southeastern Madagascar (electronic supplementary material, table S2; figure 1). Below, we show that *M. ganzhorni* diverged very recently from the Andohahela area *murinus* populations, while a much deeper split occurs between western and other southeastern Madagascar populations, all of which continue to be classified as *murinus.* Therefore, we here include *M. ganzhorni* under the nomer ‘*M. murinus s.l.*’.

We used the following geographically defined population groupings for analyses where individuals are assigned to predefined groups (figure 1): western *griseorufus* (abbreviated ‘*gri*-W’), central/contact zone area *griseorufus* (abbreviated ‘*gri*-C*’*), western *murinus* (abbreviated ‘*mur*-W’), central/contact zone area *murinus* (abbreviated ‘*mur*-C*’*) and eastern *murinus s.l.* (abbreviated ‘*mur*-E’; this population corresponds to *M. ganzhorni sensu* Hotaling *et al*. [23]).

### (b) Sequencing and genotyping

We prepared RADseq libraries following the protocol of Ali *et al*. [24]. Libraries were sequenced using paired-end 150 bp sequencing on an Illumina HiSeq 4000 at Duke University's Center for Genomic and Computational Biology sequencing facility.

After read flipping, demultiplexing, trimming and mapping to the *M. murinus* reference genome (‘Mmurinus 3.0’, [25]), we performed genotype calling with GATK v. 4.0.7.0 [26], and we filtered SNPs and individuals largely according to the ‘FS6’ filter of O'Leary *et al*. [27] (see the electronic supplementary material for details).

For the set of individuals from the contact zone area, we additionally produced two datasets using more lenient filtering procedures to be able to examine admixture using more individuals and SNPs: (i) a dataset produced by omitting the last round of removal of SNPs and individuals based on missing data; and (ii) a dataset produced using the FS6 filter without the individual-filtering steps that retained two additional putative hybrids and two individuals with mitonuclear discordance.

Based on GATK-called genotypes, we also produced full-sequence FASTA files for each RAD locus (see the electronic supplementary material for details).

### (c) Detection of hybrids using clustering approaches

For the detection of admixed individuals, we used complementary model-free and model-based approaches. First, we used principal component analysis (PCA) as implemented in the SNPRelate R package v. 1.17.2 [28], using the snpgdsPCA() function. Second, we used the program Admixture v. 1.3.0 [29] to detect clusters and assign individual-level ancestry proportions from each cluster. Third, we used the program NewHybrids v. 1.1 [21], which identified the majority of admixed individuals in Hapke *et al*. [10] and Lüdemann [11]. NewHybrids was used to estimate, for each sample, the posterior probability of it belonging to each of six predefined categories: *griseorufus*, *murinus*, F_1_ hybrid (*griseorufus* × *murinus*), F_2_ hybrid (F_1_ x F_1_), *griseorufus* backcross (F_1_ x *griseorufus*) and *murinus* backcross (F_1_ x *murinus*). Five hundred thousand iterations were used as burn-in, with another 1 500 000 iterations after that, using Jaffereys-like priors. A run was considered successful if it passed a test for convergence implemented in the hybriddetective R package [30].

### (d) Reanalysis of microsatellite data

We reanalysed the Hapke *et al*. [10] and Lüdemann [11] microsatellite data using only the samples included in this study. Like in Hapke *et al*. [10], we used the Bayesian classification methods Structure v. 2.3.4 ([19]; see the electronic supplementary material for details) and NewHybrids v. 1.1 to detect hybrids. For Structure, 20 runs using *K* = 2 were used to calculate the average membership coefficients by creating an optimal alignment using the full-search algorithm implemented in Clumpp v. 1.1.2 [31]. To keep the results directly comparable with Hapke *et al*. [10], we used the same threshold for the detection of hybrids: a sample was considered a hybrid when the posterior probability for assignment to the species of their mitochondrial haplotype was ≤0.9 for Structure or ≤0.5 in NewHybrids, and part of a specific hybrid category when the corresponding probability was greater than 0.5.

### (e) Comparison of microsatellites and single nucleotide polymorphisms using simulations

Using simulations, we compared the performance of microsatellites and SNPs for detecting hybrids. The hybriddetective R package [30] was used to generate multi-generational hybrids from both the microsatellite and SNP data. First, unadmixed *murinus* and *griseorufus* individuals were created by randomly drawing two alleles per locus from the allopatric reference populations, without replacement. For subsequent F_1_ samples, one allele per locus was drawn from an unadmixed individual of each species. This procedure, drawing from the appropriate population, was continued for F_2_ and backcross individuals. In total, 60 simulated individuals were created: 20 each of unadmixed *griseorufus* and *murinus*, and five each of F_1_, F_2_, F_1_ x unadmixed *griseorufus*, and F_1_ x unadmixed *griseorufus*. Ancestry assignment was compared between microsatellites and SNPs by running Structure and NewHybrids, as described above, on the simulated genotypes.

### (f) Phylogenetic inference

To enable subsequent tests of gene flow and demographic modelling, we determined relationships among all *murinus s.l.* and *griseorufus* individuals sampled by our study, using three *M. rufus* individuals as an outgroup. First, we used the NeighborNet method implemented in SplitsTree v. 4.14.4 [32]. This method visually displays phylogenetic conflict in an unrooted tree and thus shows phylogenetic relationships while also allowing for the detection of potentially admixed populations and individuals. Second, we used TreeMix v. 1.13 [33] to estimate relationships among predefined populations (*gri*-W, *gri*-C, *mur*-W, *mur*-C and *mur*-E) both with and without admixture events among populations.

### (g) Formal admixture statistics

The *D*-statistic and related formal statistics for admixture use phylogenetic invariants to infer post-divergence gene flow between non-sister populations. We used the qpDstat and F4RatioTest programs of admixtools v. 4.1 [34] to compute four-taxon *D*-statistics and *f*_4_-ratio tests, respectively, to test for gene flow among the predefined mouse lemur populations. For all tests, *M. rufus* was used as the outgroup. Significance of *D*-values was determined using the default *Z*-value reported by qpDstat, which uses weighted block jackknifing.

### (h) Demographic modelling

We ran the coalescent-based approaches implemented in G-PhoCS v. 1.3 [35] and BPP v. 4.2 [36], using Markov chain Monte Carlo to jointly infer population sizes, divergence times and migration rates for the three *murinus* populations (*mur*-W, *mur*-C and *mur*-SE) and the two *griseorufus* populations (*gri*-W and *gri*-SE). While G-PhoCS implements an isolation-with-migration model with continuous gene flow during potentially long periods, the multispecies-coalescent-with-introgression model in BPP models discrete introgression events.

As input for G-PhoCS and BPP, we created full-sequence FASTA files with loci for three individuals per population based on the GATK genotypes (see the electronic supplementary material for details).

We converted the migration rate parameter m to the population migration rate (2 Nm), which is the number of haploid genomes (i.e. twice the number of migrants) in the source population that arrive each generation by migration from the target population. Divergence times, population sizes and the proportion of migrants per generation (m × *μ*) were converted using empirical estimates of the mutation rate (1.52 × 10^−8^, [37]) and generation time. For the generation time, we used a lognormal distribution with a mean of ln(3.5) and a standard deviation of ln(1.16) based on two available estimates for *Microcebus* (4.5 years from [38] and 2.5 years from [39]).

## 3. Results

### (a) Genotyping

GATK genotyping followed by the standard (FS6) filtering procedure for all individuals resulted in a VCF file with 83 individuals and 60 460 SNPs. The equivalent VCF file with only samples from sympatric and parapatric sites in the contact zone area (Andahohela area, figure 1) contained 69 individuals, 12 of which were putative hybrids, and 7180 SNPs. The two less stringent filtering procedures (see Methods) for the contact zone set resulted in the retention of 78 individuals (13 putative hybrids) and 48 556 SNPs, and 79 individuals (18 putative hybrids) and 1360 SNPs, respectively. Sixteen individuals, among which two putative hybrids, did not survive the filtering steps for any of the final VCF files. The full-sequence FASTA file produced for G-PhoCS analyses contained 12 952 loci with an average length of 475 bp. For a comparison of quality control and filtering statistics among populations, see the electronic supplementary material.

### (b) No evidence for ongoing hybridization in the contact zone

**Figure 2.**
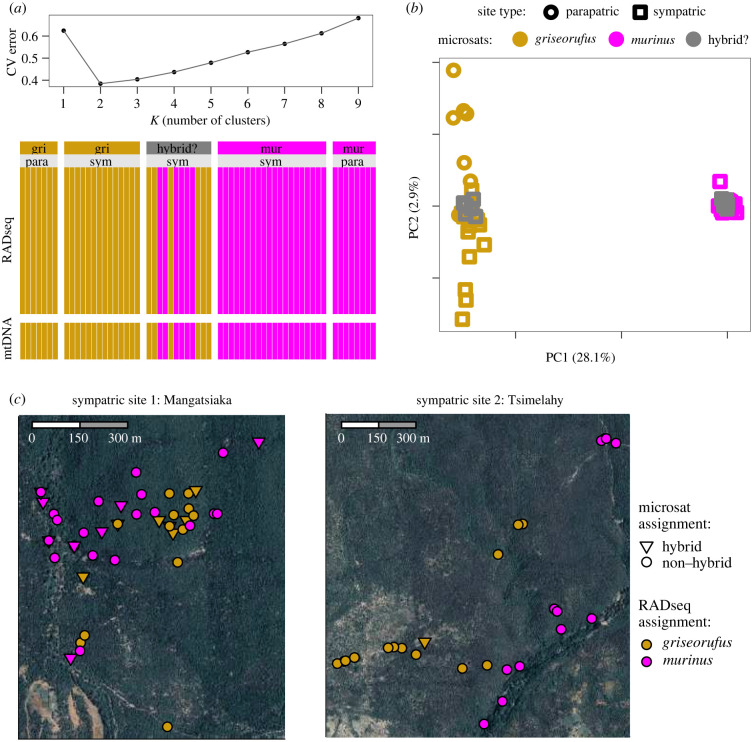
No evidence for hybridization in the contact zone. Nuclear RADseq data from the contact zone area was used for all analyses, including 12 individuals that had been identified as admixed in a previous microsatellite study (dark grey in panels *a* and *b*). (*a*) Admixture results. Top: a cross-validation error plot identifies *K* = 2 as the optimal number of clusters. Bottom: ancestry components for each individual for *K* = 2 reveal a lack of admixture: all individuals were inferred to have 100% ancestry from only a single species. Individuals were previously characterized using mitochondrial DNA (mtDNA) (bottom bars) and microsatellites (labels at top). (*b*) A PCA analysis reveals two clusters that are well-separated along PC1, corresponding to *griseorufus* and *murinus*, with no individuals that are intermediate along this axis. (*c*) Map showing spatial distribution of *murinus* and *griseorufus* individuals at the two contact sites. (Online version in colour.)

Admixture identified *K* = 2 as the optimal number of clusters among individuals from the contact zone area (figure 2*a* - top). All individuals, including the 12 putative hybrids that passed filtering, were entirely assigned to one of the two clusters (figure 2*a* - bottom), with no signs of admixture. Results were also plotted for *K* = 3, for which a third cluster corresponded to differentiation between sympatric (Mangatsiaka, Tsimelahy) and parapatric (Hazofotsy) sites in *griseorufus* (electronic supplementary material, figure S11).

PCA with individuals from the contact zone revealed a wide separation between two groups along the first principal component axis (PC1), which explained around tenfold more of the variation compared to PC2. The separation along PC1 corresponded to differentiation between *griseorufus* and *murinus,* and importantly, all putative hybrids fell within one of those two groups, with none occupying an intermediate position (figure 2*b*). Similar to the Admixture results at *K* = 3, PC2 mostly corresponded to differentiation between sympatric and parapatric sites in *griseorufus* (see also the electronic supplementary material, figure S12 for a within-species PCA).

NewHybrids was run with and without assigning individuals from the parapatric populations to reference parental species, and in both cases, all individuals were assigned to one of the two parental species and none were assigned to one of the hybrid categories. Assignment to species matched perfectly with Admixture assignments and PCA results.

Datasets produced by less stringent filtering procedures included an additional four putative hybrids that did not pass all filtering steps but could still be assessed using a more limited number of SNPs (electronic supplementary material, figure S13). Admixture and NewHybrids analyses of these datasets similarly showed no evidence for admixed individuals with the exception of mitonuclear discordance: for two of the individuals for which Lüdemann [11] had detected *griseorufus* ancestry in nuclear DNA but *murinus* mitochondrial DNA haplotypes mitonuclear discordance, we could confirm that the nuclear DNA has pure *griseorufus* ancestry (electronic supplementary material, figure S13). The third sample for which Lüdemann [11] detected mitonuclear discordance did not pass filtering at all. No other cases of mitonuclear discordance were found (figure 2*a*; electronic supplementary material, table S1.)

### (c) False positives in hybrid detection using microsatellites with NewHybrids

**Figure 3.**
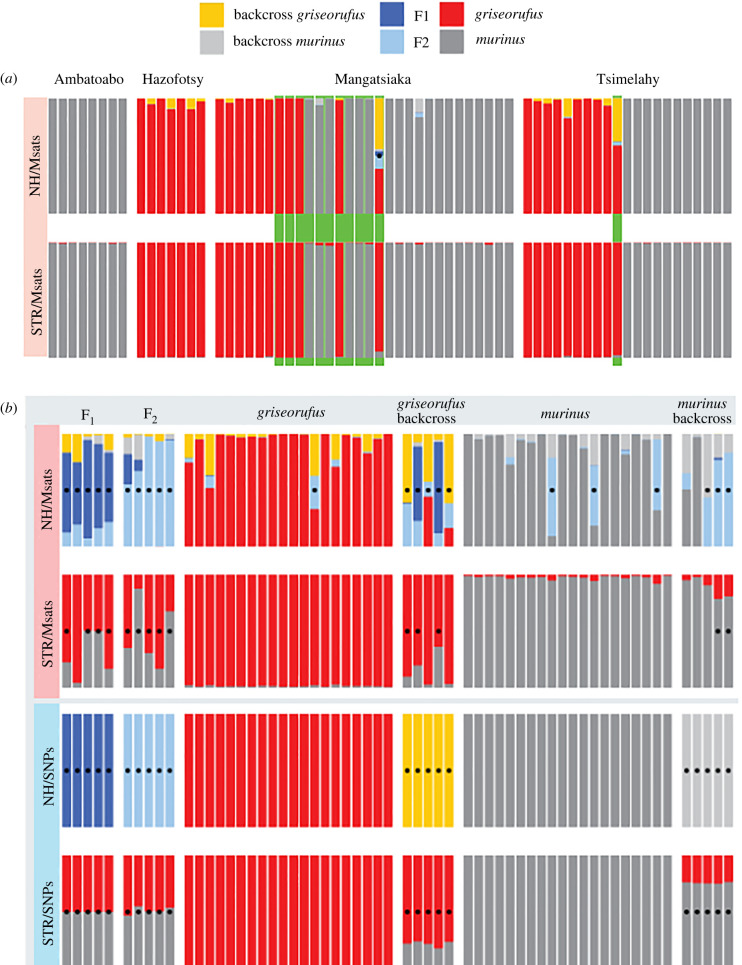
Re-analysis of microsatellite data and analysis of simulated individuals. (*a*) Re-analysis of microsatellite data with NewHybrids (NH; top row) and Structure (STR; bottom row). Among the 12 individuals previously identified as hybrids (green background bars), NewHybrids now identifies only a single individual as a hybrid (black dot), with several further *griseorufus* individuals showing non-significant signs of admixed ancestry (yellow ancestry). (*b*) Analysis of simulated individuals. Dots indicate detected hybrids. Using SNPs (bottom two rows), both NewHybrids and Structure correctly inferred ancestry for all individuals. Using microsatellites (top two rows), NewHybrids was prone to falsely inferring hybrids (4 out of 40 unadmixed individuals), and false negatives occurred both with NewHybrids (2 out of 20) and Structure (6 out of 20). (Online version in colour.)

In a reanalysis of the Hapke *et al*. [10] microsatellite data for only the individuals that were included in this study, 11 individuals identified as hybrids in Hapke *et al*. [10] were no longer identified as such by either NewHybrids or Structure. Only a single sample was now identified as a hybrid by NewHybrids, but Structure did not support this inference (figure 3*a*; electronic supplementary material, figure S14). As noted above, admixture was not detected for any individuals in the RADseq data, including those that had been identified as hybrids in the original microsatellite analyses.

**Figure 4.**
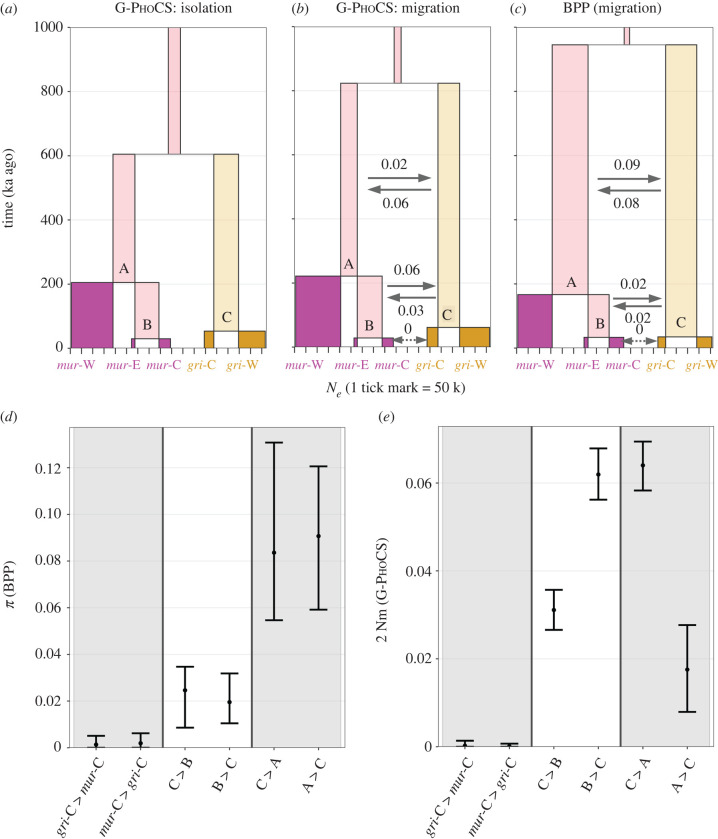
Demographic inferences using G-PhoCS and BPP. (*a–c*) Summary of results for G-PhoCS models without (*a*) and with (*b*) gene flow and for BPP (*c*; with gene flow). Each box represents an extant (bright colours: gold for *griseorufus*, purple for *murinus*) or ancestral (faded colours) lineage, with box width indicating *N_e_* and box height indicating time. Gene flow was estimated reciprocally between three pairs of lineages, as depicted by the arrows (using the same units as panels *d* and *e*). (*d*) Point estimates and 95% highest posterior densities (HPDs) of BPP introgression probabilities (*phi*). (*e*) Point estimates and 95% HPDs of G-PhoCS population migration rates (2Nm). (Online version in colour.)

In analyses of simulated microsatellite data, NewHybrids inferred that 4 out of 40 unadmixed individuals were hybrids, whereas Structure found no false positives. False negatives occurred with both NewHybrids (2 out of 20) and Structure (6 out of 20) for microsatellite data. On the other hand, NewHybrids and Structure analyses of simulated RADseq data were 100% accurate in inferring ancestry (figure 3*b*; electronic supplementary material, figure S15).

### (d) Phylogenetic approaches clarify relationships within *murinus*

A SplitsTree NeighborNet phylogenetic network (electronic supplementary material, figure S16A) of the SNP data showed a very clear separation between *griseorufus* and *murinus* with little phylogenetic conflict, and strong intraspecific structure in *murinus.* All putative hybrids fell squarely within one of the two clades, with individual assignments in perfect agreement with clustering approaches. Similarly, a NeighborNet network using only contact zone individuals showed little to no phylogenetic conflict (electronic supplementary material, figure S17).

TreeMix (electronic supplementary material, figure S16B) was run with *murinus* and *griseorufus* individuals assigned to the five populations and *M. rufus* as the outgroup, and confirmed the relationships within *murinus* suggested by SplitsTree: *mur*-W was the most divergent and *mur*-C and *mur*-E were sisters. No significant migration edges were found between *murinus* and *griseorufus,* with instead several significant edges between *M. rufus* and *griseorufus,* and *M. rufus* and *murinus* (electronic supplementary material, figure S18)*.* When *M. rufus* was excluded, significant migration edges between *griseorufus* and *murinus* did emerge, but did not include any between contact zone area populations (*gri*-C and *mur*-C) (electronic supplementary material, figure S19).

### (e) No current—but some ancestral—interspecific gene flow

*D*-statistics showed an over-representation of shared derived sites between both *griseorufus* populations (*gri*-W and *gri*-C) and the two southeastern *murinus* populations (*mur*-C and *mur*-E; relative to their sister *mur*-W, western *murinus*) (electronic supplementary material, figure S20A). Values of *D* were highly similar regardless of which of the *griseorufus* or southeastern *murinus* populations were used, which suggests historical admixture between the ancestral *griseorufus* and southeastern *murinus* lineages, as well as a lack of ongoing gene flow in the contact zone. A lack of ongoing gene flow was further supported by values of *D* very close to (and not significantly different from) zero for comparisons testing for excess derived allele sharing between contact zone populations of both species relative to their sister populations (electronic supplementary material, figure S20A).

*f*_4_-ratio tests similarly indicated ancestral admixture between *griseorufus* and the ancestor of contact zone (*mur*-C) and eastern *murinus* (*mur*-E) populations, specifically estimating that after divergence from western *murinus*, this ancestral southeastern *murinus* population experienced about 4.0–4.4% admixture with *griseorufus* (electronic supplementary material, figure S20B).

Demographic modelling using G-PhoCS and BPP supported the presence of non-zero but low levels of historical gene flow between ancestral *murinus* and *griseorufus* populations, but a lack of gene flow between extant contact zone area populations of *griseorufus* and *murinus* (figure 4*a,b*).

## 4. Discussion

We re-examined a contact zone between two species of mouse lemur in southeastern Madagascar, where significant hybridization had previously been reported based primarily on evidence from microsatellite data [10]. With RADseq data, we found no evidence for the presence of admixed individuals, and using simulations and re-analyses of microsatellite data, we showed that previously detected hybrids were probably false positives. By including allopatric populations and performing multispecies coalescent analyses, we furthermore found a general lack of ongoing gene flow, and very low levels of ancestral gene flow, between these two species.

### (a) Reconciling the lack of evidence for hybrids with microsatellite results

We found no admixed nuclear ancestry in any of the individuals from the contact zone. Our RADseq data are expected to have high power in species assignment and hybrid detection, given the combination of the relatively high number of genetic markers used [40,41] and the pronounced genetic differentiation between these two species (estimated divergence time in a no-migration scenario: approximately 600 ka ago; figure 4; average *F*_ST_ in the contact zone area: 0.40; electronic supplementary material, table S5). Furthermore, in a re-analysis of microsatellite data using the same methods as the original studies [10,11], though restricted to the individuals used in this study, all but one of the previously detected hybrids were no longer classified as such (figure 3*a*).

Considering the clear and robust RADseq results, it is highly unlikely that true hybrids were missed in our analyses. Instead, our results suggest that the hybrids inferred in Hapke *et al*. [10] were false positives, and more generally, that the inference of hybridization using microsatellites can be sensitive to such false positives, particularly when using the program NewHybrids.

In our simulations with microsatellites, Structure suffered from false negatives only, whereas NewHybrids produced four false positives among 40 simulated unadmixed individuals (figure 3*b*). Additionally, in our reanalysis of the microsatellite data, the single individual that NewHybrids continued to assign hybrid ancestry to did not show signs of admixture using Structure (figure 3*a*). In Hapke *et al*. [10], their fig. 5), Structure did not consistently infer admixed ancestry for several of the putative hybrids. This was especially apparent when parapatric populations were included, in which case only four out of the 12 NewHybrids positives showed admixed ancestry using Structure (and three out of those four were still assigned less than 10% admixed ancestry by Structure, [10], their fig. 5). Even though NewHybrids appears considerably more prone to false positives than Structure, the latter did show admixed ancestry for sevenindividuals in an analysis using only individuals from the contact zone site Mangatsiaka (versus nine with NewHybrids). At the same time, both programs had 100% accurate assignments with simulated SNP data, suggesting that the false positives found in the microsatellite analysis stem mostly from challenges with this type of molecular marker, to which NewHybrids appears to be more sensitive than Structure.

### (b) Evolutionary resolution of microsatellite versus single nucleotide polymorphism data

The results of our simulation analysis suggest that microsatellite data are vulnerable to both false positive and false negative detection of admixture between species. This effect will be especially significant when parental lineages are sufficiently phylogenetically diverged such that the rate of recurrent or backward mutation will obscure the true evolutionary signal [2,4]. To our knowledge, this study is the first to directly compare microsatellite and SNP data in a population genetic analysis within mammals. As reviewed by Sunde *et al*. [42], such ‘head-to-head’ studies are extremely rare and are presently limited to plants and fishes. Nonetheless, relative strengths and weaknesses of the two data types are emerging. Whereas earlier assessments of microsatellite data posited that their extremely high evolutionary rate would make them ideal for revealing subtle population genetic parameters [4,7], direct comparison with SNP data is showing the opposite to be true. Indeed, these studies indicate that SNP data are more sensitive across a broad range of evolutionary parameters, including phylogenetic structure, admixture, population subdivision and measures of heterozygosity [42–44]. Recent work is also clarifying the degree to which SNP data are robust to small organismal datasets, even those with as few as *n* = 2 [44]. These observations and assessments are further supported by both the simulation and empirical results reported in this study.

### (c) Lack of ongoing gene flow and implications for speciation

The presence of at least two individuals with mitonuclear discordance (a *griseorufus*-type mitochondrial haplotype, and *murinus* nuclear DNA) may suggest some ongoing or recent gene flow between the two species. However, consistent with the lack of evidence for nuclear admixture in contact zone sites, we found no evidence for ongoing gene flow using multiple methods, including a phylogenetic network (electronic supplementary material, figure S16A), TreeMix (electronic supplementary material, figure S16B), formal admixture statistics (electronic supplementary material, figure S20) and two multispecies coalescent methods (G-PhoCS and BPP; figure 4*f*). Combined with syntopic occurrence in at least one of the contact zone sites (figure 2), these findings strongly suggest that *murinus* and *griseorufus* are currently reproductively isolated, which is striking giving the estimated divergence time of less than 1 Myr (see also [45]).

Little is known about the relative importance of different types of reproductive isolation in mouse lemurs. Across their ranges, *murinus* and *griseorufus* occur in distinct habitat types, with *griseorufus* mostly limited to spiny forests that appear to be too arid for *murinus* [46,47]. Separation by habitat (e.g. [48]) at larger scales could therefore minimize or even prevent syntopic co-occurrence despite nominal sympatry in the contact zone area, thus limiting interactions between the species. At one of the two sympatric sites included in this study, Tsimelahy, species-specific sampling locations are indeed consistent with separation by habitat, but at the other, Mangatsiaka, the two species co-occur even at a very fine spatial scale ([46]; figure 2*c*). Therefore, the observed lack of gene flow is unlikely to simply be a by-product of separation by habitat, and additional sources of pre- and/or postzygotic reproductive isolation need to be invoked.

## 5. Conclusion

Using RADseq data, we found no evidence for admixture between two species of mouse lemurs in a contact zone in southern Madagascar. This is in sharp contrast to a previous study that found widespread hybridization among the same samples using microsatellites. Our results suggest that the hybrids inferred by the previous study were probably false positives, and we urge caution when using microsatellites to infer hybridization. Thus, our results support concerns around the usage of microsatellites—most importantly, that rates of evolution in microsatellites are simply too high for use at interspecific levels given their propensity for homoplasy beyond the intrapopulation level [7,49]. Finally, we estimate a divergence time of less than 1 Myr and a lack of historical gene flow, which in combination with local syntopic occurrence and no evidence for admixture, suggests the rapid development of reproductive isolation between these species.

## Data Availability

Sample metadata can be found in the electronic supplementary material, table S1. Additional metadata and processed data, such as VCF files and analysis input and output files can be found at the Dryad Digital Repository at https://doi.org/10.5061/dryad.1jwstqjx3 [50]. All code used to run the analyses and produce the figures in this manuscript can be found on GitHub at https://github.com/jelmerp/lemurs_contactzone_grimur. Raw sequence data is available through the NCBI (Bioproject PRJNA861727). Data are provided in the electronic supplementary material [51].
